# Lipid and Protein Oxidation Marker Compounds in Horse Meat Determined by MIR Spectroscopy

**DOI:** 10.3390/foods9121828

**Published:** 2020-12-09

**Authors:** Irati Jáuregui-López, Fernando Zulategi, María José Beriain, María Victoria Sarriés, Miguel Beruete, Kizkitza Insausti

**Affiliations:** 1 Antennas Group-TERALAB, Campus de Arrosadía, Universidad Pública de Navarra (UPNA), 31006 Pamplona, Spain; irati.jauregui@unavarra.es (I.J.-L.); miguel.beruete@unavarra.es (M.B.); 2 Multispectral Biosensing Group, Navarrabiomed, Complejo Hospitalario de Navarra (CHN), Instituto de Investigación Sanitaria de Navarra (IdiSNA), Universidad Pública de Navarra (UPNA), Irunlarrea 3, 31008 Pamplona, Spain; 3 Research Institute for Innovation and Sustainable Development in Food Chain (IS-FOOD), Campus de Arrosadía, Universidad Pública de Navarra, 31006 Pamplona, Spain; zulategui.79089@e.unavarra.es (F.Z.); mjberiain@unavarra.es (M.J.B.); vsarries@unavarra.es (M.V.S.)

**Keywords:** MIR (Mid-Infrared) spectroscopy, lipid oxidation markers, protein oxidation markers, foal meat

## Abstract

This work broadens the study of lipid and protein oxidation marker compounds in foal meat, employing the technology of Attenuated Total Reflectance-Fourier Transform Mid-Infrared Spectroscopy (ATR-FT/MIR, shortened in the following as MIR). As a main objective, marker compounds from 23 foals were extracted and their absorbance spectra were measured to establish prediction models (calibration and validation) between them and classical quantification analysis of the compounds. Another objective was to ascertain whether a previous extraction of the marker compounds before executing their MIR analysis is preferable compared to direct MIR measurements on the raw meat samples. In this context, marker compound results (TBARS between 0.4387 and 2.1040, and carbonyls between 4.07 and 4.68) showed more consistent predictive models than the ones achieved using quantitative analysis of the spectra obtained from the raw meat. Lipid oxidation compounds predictive models obtained in this work offered an R^2^_cv_ of 63.18% and protein oxidation R^2^_cv_ obtained in this project showed a value of 54.24%. Thus, MIR technology arises as a promising tool to identify and quantify products derived from lipid and protein oxidation in fresh foal meat.

## 1. Introduction

While historically horse breeds used for consumption came from old animals previously employed for field labor, nowadays these animals come from selected breeds with meat production purposes [1]. Around 700,000 foals graze in Spanish lands [2] and thousands of livestock heads are slaughtered annually, e.g., 38,200 during the year 2019, according to the data provided by the Ministry of Agriculture, Fisheries and Food of the Spanish Government [3]. Thus, Spain is positioned as an important foal meat exporter and it is one of the biggest European producers. Foals raised for commercialization are animals between 7 and 9 months of slaughter age (*lechales* foals), or between 15 to 16 months of slaughtering age (*quincenos* foals) [4], demystifying the extended belief that horse meat always comes from old and rejected animals employed for other purposes. 

Foal meat composition presents several differences with other types of meat. Its main peculiarity is its higher protein content and lower fat content than other popular meats. Concretely, foal meat presents a 2% of total lipids in its composition, 32% of which are saturated fatty acids (SFA), while lean beef has up to 5.4% of fat, of which 41% are SFA [5]. In the last years, several works and results about foal meat’s lipid composition can be found in the literature and it has been observed that, regarding fatty acids (FA) in intramuscular fat foal muscles, the most predominant ones are Polyunsaturated Fatty Acids (PUFA), ranging from 41.1 to 48.2% of the total methyl esters [6]. With respect to proteins, foal meat is characterized by having a higher proportion of protein content than beef or pork, and with a high biological value, as it presents around 40% of essential amino acids [7].

Oxidation is one of the main causes, if not the principal, of meat quality loss during the meat preservation process as it affects lipids, proteins, carbohydrates and vitamins in meat, producing changes in the meat consistency, texture, color and sensory properties in general. During the aging and storage time, the close relationship between lipid and protein oxidation (L_OX, P_OX) in fresh meat and its influence on the deterioration of meat quality is already known [8]. Over the years, L_OX has been the subject of plenty of studies, leading to a substantial knowledge of the principal mechanisms involved in PUFA oxidation and parallel degradation. The oxidation process involves the degradation of PUFA or vitamins, among others, giving rise to the generation of free radicals, more susceptible to suffer oxidation, leading to changes in the meat composition and its nutritional value [9]. L_OX is a convoluted mechanism that involves complex chains of reactions that gives rise to different primary (peroxides), and secondary oxidation compounds [10]. Associated changes to L_OX constitute the principal cause of degradation in meat and meat products, as they trigger bad odors and flavors formation, as well as color alteration, all of it consequently leading to a loss in the organoleptic quality of the final product. In addition, this L_OX also leads to a loss in the meat’s nutritional value, and to the formation of potentially harmful compounds related to different pathologies [11]. 

L_OX, through the oxidant activity of primary and secondary L_OX products, is also thought to promote the oxidation and degradation of proteins. According to Pignoli et al. [11,12], the different chemical modifications in specific amino acid chains, or the peptide backbone, can lead to changes in the physical properties of the proteins, with different chemical manifestations, such as gain of carbonyl derivatives, formation of intra and intermolecular cross-links, or loss of tryptophan fluorescence, among others. Color changes in foal meat occur more quickly than in other meats such as beef due to a higher iron content, which affects the myoglobin [13]. Besides, tenderness is usually greatly affected by P_OX. Meat becomes harder and less juicy due to changes in protein cross-linking, due to oxidation reactions [14]. P_OX is mainly produced due to the presence of free radicals that act as oxidizing agents. These oxidation agents act on the side chains of amino acids, causing the loss of the amino group, and the formation of carbonyl groups. In addition, L_OX usually has a direct influence in P_OX. Nevertheless, the protein degradation can also occur without L_OX. The carbonyl formation is also favored by refrigerated storage and freezing of the meat, as well as by culinary treatments. Contrary to what happens to L_OX, protein degradation has not been as deeply studied in foal meat quality researches [15], despite having been proved that P_OX has an important effect on the functional properties of meat, and on foal meat in particular [12]. 

In this context, Lipid/Carbonyl radicals, hydroperoxides and malonaldheyde (MDA) play a vital role in promoting in vivo oxidative reactions, which have been proven to not only reduce the quality of meat and food but also to increase health risks. That is why through the years different techniques have been developed in order to detect and measure some of these compounds. Concretely, quantification of milligrams of MDA per kilogram of meat sample by the Thiobarbituric Acid Reactive Substances (TBARS) analysis; and quantification of the total amount of carbonyls by using the Dinitrophenylhydrazine (DNPH) technique are frequent methods for assessing L_OX and P_OX, respectively. These techniques are time consuming, though. Thus, there is an increasing interest in the food industry on the application of new, fast, and reliable techniques. In this way, Attenuated Total Reflectance-Fourier Transform Mid-Infrared Spectroscopy (ATR-FT/MIR, shortened in the following as MIR) might be a practical option. MIR spectroscopy applications in food analysis are diverse, although its current use is limited [16,17]. In this context, the objective of this research was to study the usefulness of MIR technology to estimate the values of L_OX and P_OX marker compounds in foal meat, due to its high susceptibility to oxidation. To do so, MIR spectra were recorded on L_OX and P_OX marker compounds previously extracted from raw meat samples. Then, a second objective, was to study if MIR technology is more useful when applied on previously extracted oxidation compounds or when measured directly on raw meat samples.

## 2. Material and Methods

### 2.1. Animal Management and Meat Sampling

In this study, two different types of animals (differing in age and feeding diet) and four meat aging times were used in order to obtain samples with potentially different oxidation levels. 

Foals were kept with their mothers and allowed to suck freely on the pasture from birth to weaning at the age of 6 to 7 months. Then, foals were randomly divided in two groups to be slaughtered at two different ages: 12 foals were slaughtered at 26 months of age (784 ± 37 days) which correspond to the “Adult” (A) group. The diet (Appendix A) of the A group was supplemented with a standard/conventional concentrate (C). Then, 11 foals were slaughtered at 13 months (403 ± 30 days), which corresponds to the “Young” (Y) group, whose diet was supplemented with a linseed-rich concentrate (L) (5%). This fattening period lasted around 104 days (±10 days) in both groups.

Foals were transported 50 km to the abattoir the day before slaughter in compliance with current European regulations (Council Regulation 1/2005EC, 2005), and were stunned with a captive bolt, slaughtered, and dressed according to the specifications outlined in the European legislation (Council Directive 93/119/EC, 1993). Twenty-four h post-mortem, Longissimus dorsi (LD) muscles of each of the animals were extracted from the left half-carcasses. The LD muscle was sliced into 20 mm (±0.2) thick cuts, and steaks were aged under vacuum (99%) and in refrigerated chambers at 2 ± 1 °C and under dark during the corresponding days (0, 4, 8, or 12 days for the Y-L group; and 4 days for the A-C group). Once these periods elapsed, samples were frozen during 3 months (±10 days). After that, samples were thawed during 24 h under refrigerated conditions (2 ± 1°C) before analysis. Meat from the A-C group was aged 4 days, as this is the standard aging time for this type of meat. Meat from the Y-L group was aged longer in order to promote potential oxidation of the meat due to the linseed diet fed to the foals. 

### 2.2. Experimental Design

**Figure 1:**
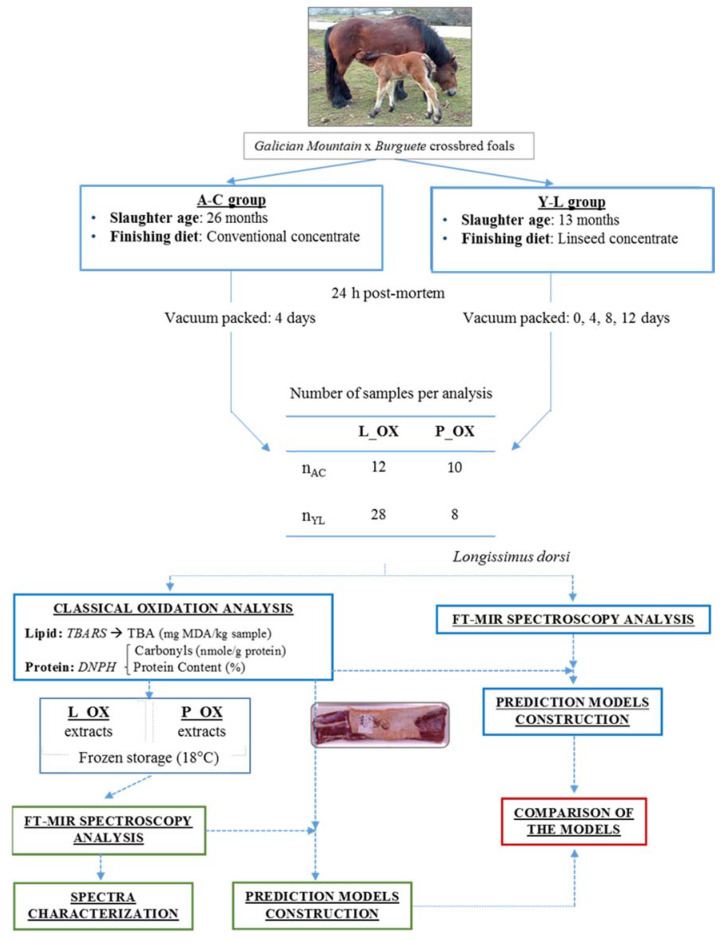
Scheme of the experimental design for both models performed in this work (n_AC_: number adults on concentrate; n_YL_: number youngs on linseed; TBARS: Thiobarbituric Acid Reactive Substances; DNPH: Dinitrophenylhydrazine; L_OX: lipid oxidation; P_OX: protein oxidation).

Once the meat cuts from each animal were fridge-stored for the time required in the analysis, two different parts in the design were performed according to two analyses: L_OX quantification by TBARS and MIR spectroscopy analysis; and P_OX quantification by DNPH method and MIR spectroscopy analysis. In both L_OX and P_OX analysis, MIR spectra were recorded to previously extracted oxidation compounds, and directly on raw meat samples. A scheme of the experimental design is shown in Figure 1.

### 2.3. Analytic Method for Both Lipid and Protein Oxidation Quantification

#### 2.3.1. Lipid Oxidation Quantification: Thiobarbituric Acid Reactive Substances (TBARS) Analysis

L_OX was evaluated through the method proposed by [18], with some variations applied. Two grams of meat were minced and 10 mL of 5% trichloroacetic acid (TCA) was added. Then, it was homogenized with an Ultra-Turrax (IKA T25 digital ULTRA-TURRAX, IKA-Werke GmbH & Co., Staufen im Breisgau, Germany) for 1 min at 11,500 rpm. The homogenized sample was stored at −10 °C for 10 min and was centrifuged at 5000 rpm, for 10 min at 4 °C. Supernatant was filtered using a Filter-lab no 1246 filter on ice. One mL of the filtered substance was taken and reacted with 1 mL 0.02 M TBA. Immediately, it was incubated in a water bath at 96 °C for 40 min. Then, tubes were chilled and centrifugated at 10,000 rpm for 4 min at 20 °C. Part of the extract was used for MIR measurement (L_OX marker compound). Absorbance was measured on the rest of the extract at 530 nm employing a UV/vis Spectrophotometer with diodes detector, model Shimadzu UV-2101PC. TBA values were calculated from a pattern curve of 1,1-3,3 tetraethoxypropane (TEP) and expressed as mg of MDA/kg of meat sample. 

#### 2.3.2. Protein Oxidation Quantification: Dinitrophenylhydrazine (DNPH) Method

P_OX, measured as total carbonyl content, were quantified according to the method described by Oliver [19] and modified by Vuorela [20], commonly known as the DNPH method. Each of the meat samples was homogenized with 20 mL of 0.6 M NaCl for 60 s using an Ultra-Turrax homogenizer (IKA T25 digital ULTRA-TURRAX, IKA-Werke GmbH & Co., Staufen im Breisgau, Germany). Two aliquots of homogenate were taken (0.1 mL) and were transferred into Eppendorf vials. Proteins were precipitated with 10% (1 mL) TCA and were centrifuged for 5 min at 10,000× *g*. One of the pellets was treated with 2N HCl (1 mL) in order to quantify proteins and the other one with 0.2% 2,4-dinitrophenyl hydrazine (DNPH) in HCl 2M (1 mL) to quantify carbonyls. Part of the extract was used for MIR measurement (P_OX marker compound). Protein concentration samples (p) were measured spectrophotometrically attending to absorbance at 280 nm (Spectrophotometer UV/vis with diode detector, Shimadzu UV-2101PC, Mettler-Toledo S.A.E. L’Hospitalet de Llobregat, Barcelona, Spain) using Bovine Serum Albumin (BSA) as the standard. Carbonyl content was expressed as nmol of carbonyl compounds per milligram of protein using an extinction coefficient of 21.0 mM^−1^ cm^−1^ at 370 nm.

### 2.4. MIR Spectra Acquisition

The measuring instrument used in this research was a FTIR Vertex 80v spectrometer (Bruker, Ettlingen, Germany). The measurements were made with an ATR Platinum (Bruker, Rheinstetten, Germany) inserted in the FTIR. The spectrometer was equipped with a Globar source (operation bandwidth, 6000–50 cm^−1^), a beam splitter of KBr (10,000–400 cm^−1^), and DLaTGS detector (10,000–250 cm^−1^). The active area of the ATR Platinum was a diamond crystal (2 × 2 mm, approximately) on top of which the samples under test were deposited.

First, a reference spectrum was taken with the ATR device empty, and then the spectrum of each of the samples was measured. The different samples were placed on the diamond crystal of the ATR, ensuring that the entire crystal surface was completely covered and that there was a perfect contact between the samples and the crystal. Once every sample measurement was performed, the ATR diamond crystal was cleaned with a piece of Berkshire Durx^®^ 670 optic cleaning wipe using distilled water and isopropyl alcohol to leave it ready for the next sample’s reference measurement. In order to stablish the adequate number of replicates, 32 scans were averaged in the 4000 to 400 cm^−1^ spectral range and were recorded with a resolution of 4 cm^−1^. It was observed that, on average, 1 out of 15 measurements of the same sample showed outlier or anomalous spectral results in comparison to the others (this was performed for a total of 105 measurements from 10 different samples). In this way, it was stablished that repetitions of the same sample would be enough to obtain consistent and similar results. Thus, taking into account the number of extracts used (see Figure 1) a total of 240 measurements were performed for L_OX determination, and a total of 108 measurements were carried out for P_OX determination. Every measurement provided a single and unique spectrum, which was saved in the PC in order to employ them for the chemometric model of the data obtained. The spectra acquisition of the raw meat samples was done and described in a previous work [21].

### 2.5. Statistical/Chemometric Treatment

The analysis of variance (ANOVA) was performed using the General Linear Model (GLM, SPSS 23.0, Chicago, IL, USA) to study the TBARS and carbonyl content of the samples. Significant differences (*p* < 0.05) between groups were tested using Tukey’s test.

In order to perform the quantitative analysis, the software package QUANT 2, from Bruker’s Opus v.7.0, was used. The purpose of QUANT 2 is to perform quantitative analysis of unknown multicomponent samples. A chemometric model, using a number of calibration samples of known composition that were representative of the studied system, was used. Outliers and negative bands were discarded and MIR spectra of the remaining samples would then be used by QUANT 2 to calculate a calibration function, which essentially is the model used for the analysis of unknown samples. 

The cross validation method was employed, which uses the same set of samples for calibration and validation. In this method, a representative set of samples for a multicomponent system is used to calibrate and validate a system. Before starting the calibration, one of the samples was excluded from the entire set of samples, and used for the validation process. The remaining samples were then used to calibrate the system. In order to do this, a set of calibration samples was measured by MIR spectroscopy. With the obtained spectral results, the main absorption peak amplitudes for each substance were determined and plotted versus different known concentrations. Thus, the resulting calibration graph was used to evaluate the concentration of an unknown sample, by measuring its absorption peaks and comparing them with the resulting graph. The information contained in the calibration samples spectra was then compared to the information of the known concentration values using a Partial Least Square (PLS) regression. This method assumes that systematic variations observed in the spectra are a consequence of the changes in the concentration of the components. 

Multivariate calibrations make use of not only a single spectral point but they take into account spectral features over a wide range. Therefore, the analysis of overlapping spectral bands or broad peaks becomes feasible. Multivariate calibrations require a large number of calibration samples and yield a large amount of data (several spectra with hundreds or thousands of relevant data points). In order to conveniently handle so many results, the spectral response and its concentration values were written in a matrix form, where each row in the spectral data matrix represented a sample spectrum, and the concentration data matrix contained the corresponding concentration values of the samples. The matrices were decomposed into their Eigenvectors, by which it is possible to define a spectral characteristic without using all its components. Thus, only the relevant principal components were taken into account, leading to a considerable reduction in the data amount. Finally, a PLS regression algorithm was deployed to find the best correlation function between spectra and concentration data matrix.

#### Data Preprocessing

In order to ensure the reproducibility of the calibration samples, several spectra of each sample were acquired. Due to the inhomogeneity of the samples, a spectral normalization was first performed. Then, data preprocessing procedures were applied to align the different spectra. Data preprocessing can eliminate variations in offset or different linear baselines and is useful to ensure a good correlation between the spectral data and the concentration values. In this study, different preprocessing methods were used for L_OX and P_OX results. When working with the marker compound samples, two preprocessing steps were applied. First, a whole calibration/validation, using the entire data set with all the studied samples, was made. Due to the fact that there were many samples, and in order to homogenize the spectral results, a second calibration/validation model with reduced data was also elaborated by taking the average of six repetitions for each sample, and were referred to as the Mean Set.

Regarding the L_OX results acquisition, two methods were employed: Standard Normal Variable Vector Normalization (SNV), which normalizes a spectrum by first calculating the average intensity value and subsequent subtraction of this value from the spectrum; and then calculates the sum of the squared intensities, and divides the spectrum by the square root of that sum; and a Linear Offset Subtraction, which shifts the spectra in order to set the absorbance minimum to zero. 

In the case of P_OX results, a combination of two methods was employed. First, a Multiplicative Scatter Correction, which performs a linear transformation of each spectrum for it to best match the mean spectrum of the whole set. Second, a First Derivative method, which calculates the first derivative of the spectrum, emphasizing the steep edges of a peak, was employed to obtain the carbonyl content results. Finally, a Min-max Normalization method (first subtracts a linear offset and then sets the absorbance-maximum to a value of 2 by multiplication with a constant) was employed to obtain the results of the carbonyl content using the Mean Set.

As a final step, a discussion of the results obtained with the quantitative analysis of the spectra from the oxidation marker compounds (L_OX and P_OX) vs. the ones obtained in the quantitative analysis of the spectra analyzed directly from raw meat in a previous work [22] was carried out.

## 3. Results and Discussion

### 3.1. Lipid and Protein Oxidation Quantification

The L_OX and P_OX quantification was done using the analytically obtained lipid (TBA) and protein (DNPH) oxidation values from Y-L and A-C animals. As shown in Table 1, TBA content showed an increasing trend, though non-significant, with ageing time (0–12 days), meaning a higher L_OX in aged samples, as it was expected from the results found in [8]. As it can be observed in Table 1, the results for the A-C group in TBA analysis at day 4 showed higher MDA content, with 2.1 mg MDA/kg, in comparison to the 0.44 mg MDA/kg of meat obtained for the Y-L group, at an ageing time of 4 days (*p* < 0.001). These results suggest that the MDA content in foal meat increases as the slaughter age increases, most likely due to a higher intramuscular fat percentage of older animals [23]. Nevertheless, if redoing the statistical analysis by subtracting the AC group, significance in the data is still found for different days of ageing (data not shown).

According to Zakrys-Waliwander [14] and Ruiz [21], P_OX in beef samples increases with oxygen and conservation ageing time. For this reason, similar behavior was expected for P_OX of foal meat than the one obtained for TBA. Nevertheless, the results presented in Table 1 do not show any apparent increase or decrease, nor any clear pattern followed by the samples. As shown in Table 1, differences in the carbonyl content were only due to the differences observed between the A-C group and the Y-L-0d group (*p* < 0.01). This way, although it is demonstrated that TBA increases as the slaughtering age does, there is no evidence that it occurs in a similar way in the case of carbonyls. Regardless, both oxidative processes should be taken into account for a correct determination of meat quality, allowing the evaluation and differentiation of the oxidative changes that take place in lipids and proteins.

### 3.2. Spectral Characterization

**Figure 2:**
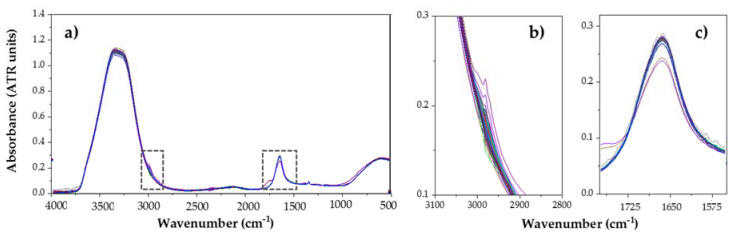
(**a**) ATR MIR spectra (Attenuated Total Reflectance-Fourier Transform Mid-Infrared Spectroscopy) of L_OX (lipid oxidation) samples (240 analyses) and expanded areas of interest: (**b**); 2800–3100 cm^−1^. (**c**) 1525–1750 cm^−1^.

**Figure 3:**
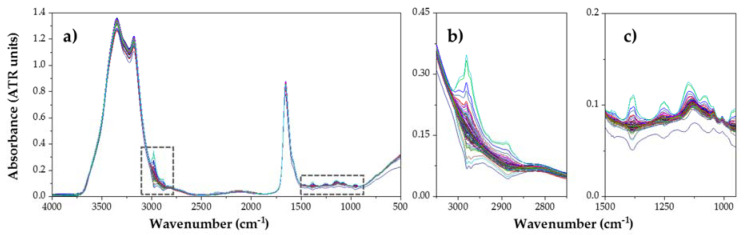
(**a**) ATR MIR spectra of P_OX (protein oxidation) samples (108 analyses) and expanded areas of interest: (**b**) 2750–3100 cm^−1^; (**c**) 950–1500 cm^−1^.

In this section, the spectral MIR characteristics of the analyzed substances are studied, with a total of 348 analyzed spectra, shown in Figure 2 and Figure 3. As it can be observed, a moderate variability is shown in L_OX and P_OX content extract samples’ spectra (see Figure 3). In order to study the different peaks present in the spectra, a bibliographic revision of the main absorption peaks of these kinds of substances was performed. Table 2 contains a compilation of the main wavenumbers associated to their functional groups according to the bibliography consulted, in relation to the compounds considered.

#### 3.2.1. Spectral Characteristics of Lipid Oxidation Marker Compounds 

**Figure 4:**
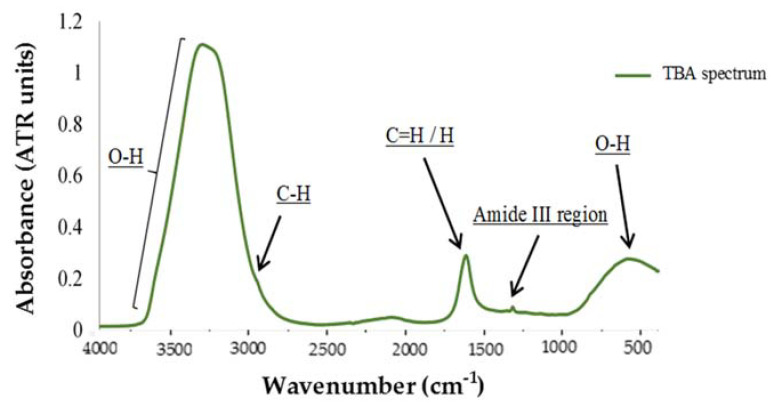
Example of MIR spectrum corresponding with L_OX (lipid oxidation) compounds.

Among all the absorption peaks shown in the Figure 4, the most significant one is the band located between 3700 cm^−1^ and 3100 cm^−1^, which corresponds to the wide band of high absorption associated to the hydroxyl groups O-H of water. In this case, it hides the possible presence of other peaks. In the same way as before, vibrational oscillations of these O-H groups provoke the apparition of another wide band between 900 cm^−1^ and 400 cm^−1^. After these two regions, the band with the highest absorption appears between 1680 cm^−1^ and 1590 cm^−1^, with a peak at 1636 cm^−1^. This area, according to [29], corresponds to the stretching vibrations of C=H groups (Alkenes). However, the peak placed at 1640 cm^−1^ corresponds to the hydrogen covalent bonds bending of water. This way, results are not clear enough to determine the type of bond that provokes that absorbance. Moreover, a weak peak can also be observed at 2981 cm^−1^, again associated to the different vibrational oscillations of C-H bonds. This one in particular was assigned to the stretch of C-H in the study of Tertiary Amine-Triethylamine [33]. Finally, it can be clearly observed that an isolated peak in the Fingerprint Region (from 1500 to 400 cm^−1^) [29] was present in each of the 240 samples measured. The peak is located at 1336 cm^−1^ and has a low but repeated absorption from 0.086 to 0.095 (mean = 0.088). No reference regarding this particular peak has been found in any of the works consulted in the literature. Nevertheless, it is located in the region correspondent to CH3 symmetric deformation and CH2 wagging band (1400–1200 cm^−1^), so it is supposed to belong to this kind of peptide complex band. As a curiosity, the only reference found regarding the apparition of a peak at this particular frequency was in the study of [34], in which they found that the band corresponding to 1336 cm^−1^ may be considered a sensitive marker for the identification of *Lactobacillus casei*, used to evaluate lactic fermentation in fermented foods production.

#### 3.2.2. Spectral Characteristics of Protein Oxidation Marker Compounds 

**Figure 5:**
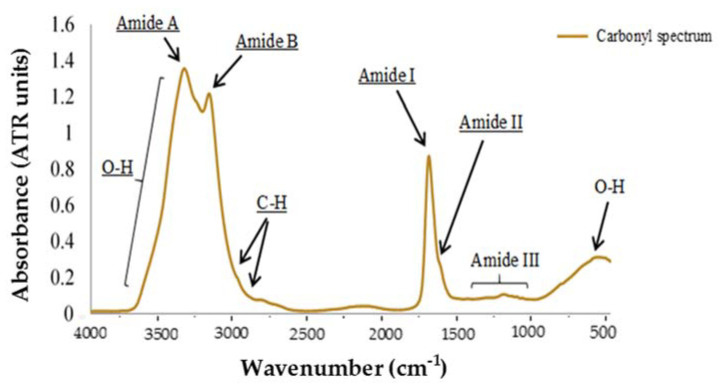
Results of MIR absorbance spectra from P_OX (protein oxidation) compounds.

Regarding MIR analysis of a carbonyl sample, two areas can be differentiated in order to perform a more detailed study (Figure 3); the Functional Group Region, between 4000 and 1500 cm^−1^, which corresponds to hydrogen bonds atoms (C-H, O-H, and N-H) vibrational oscillations; and the Fingerprint Region (1500–400 cm^−1^). Among all the absorption peaks that can be seen in the graph, the most remarkable ones are those located in the Functional Region, and more concretely between 3700 and 3100 cm^−1^. This range presents high absorption associated to the hydroxyl groups O-H, where water appears. Usually, these absorption peaks hide the N-H associated peaks [35]. However, Figure 5 shows two clearly differentiated peaks, corresponding to Amide A group, at 3350 cm^−1^; and Amide B group, at 3175 cm^−1^, which is originated from a Fermi resonance between the first overtone of Amide II and N-H stretching vibrations [25]. If we observe both graphs, there is another peak that stands out over the rest. It is found at 1650 cm^−1^ and corresponds to the Amide I band, the most intense absorption band in proteins. This is primarily due to the stretching vibrations of the C=O (70–85%) and C-N groups (10–20%), and its frequency can be found in the range between 1600 and 1700 cm^−1^. Next to Amide I group, another less pronounced peak appears between 1588 and 1565 cm^−1^ known as Amide II. This is a more complex region than Amide I, and derives mainly from in-plane N-H bending (40–60% of the potential energy). The rest of the potential energy arises from the C-N (18-40%) and the C-C stretching vibrations (about 10%) [36]. Finally, Amide III band can be found at the region between 1400 and 1200 cm^−1^, and corresponds to complex bands dependent on the details of the force field, the nature of side chains and hydrogen bonding [26].

If we observe how the weaker peaks, we find moderate absorbance at 3000 cm^−1^, associated to the C-H bonds different vibrational oscillations. The highest peak among them is the one located at 2980 cm^−1^ and corresponds to the asymmetrical stretching vibrations of Alkanes, which could represent the fatty acids backbone (carbon atom basis of fatty acids), for both methyl (-CH_3_) and methylene (-CH_2_) groups. Finally, a wide band with large absorption is observed in the region between 900 and 400 cm^−1^, which again corresponds to hydroxyl groups O-H of water, and thus they do not give any substantial structural information [24].

### 3.3. Multivariate Analysis and Data Processing

In this section, the validation and calibration models for both protein and lipid oxidation results, for the two kinds of samples studied, raw meat, and marker compounds, were performed. All of these results are shown in Table 3, where the two first columns correspond to TBA and carbonyl content of raw meat samples; whereas the rest of the columns correspond to L_OX and P_OX marker compounds. 

#### 3.3.1. Lipid Oxidation Results

As observed in Table 3, several differences can be found among the obtained results. With regard to TBA results, there are significant differences between the raw meat samples, and the ones obtained from the compounds themselves (marker compound samples), showing a calibration R^2^ of 9.96% and 75.19% respectively, which means a difference of 65.23%. Regarding the validation models, there are also similar differences between both kinds of samples, showing an R^2^_cv_ of 2.43% for raw samples; and 63.18% for the marker compounds samples. The rest of the studied parameters presented less consistent results in the raw meat samples than in the compound markers. 

In this section, a total of 240 spectral analyses from 40 L_OX samples were employed for the model construction. The samples were extracted from a total of 19 different animals from two different groups (*n* = 7 Y-L and *n* = 12 A-C). Results of the first validation were not very consistent, as they presented a poor R^2^, and a high Root Mean Square Error of cross validation (RMSECV). Because of that, some preprocessing methods were applied to the spectral data, and the most remarkable outlier points were subtracted from the cross validation in order to obtain a stronger and more reliable result. From all the possibilities, we selected the one with minimum RMSECV and higher rank and regression point displacement (RPD) values. 

The resulting validation model after the preprocessing procedures showed a R^2^ of 63.14% (33.83% without preprocessing), RMSECV of 0.402 (0.685 without preprocessing), and RPD of 1.65 (1.23 without preprocessing). In order to make the validation, we employed a cross validation model with one sample exclusion. In this case, 13 outlier spectra were subtracted from the results, so a total of 227 spectral data were employed for the calibration/validation model. The studied spectral range can be divided in two different regions: the region between 3278 and 2918 cm^−1^; and the area between 1.839 and 759 cm^−1^. The L_OX concentration suffered variations in a range between 0.28 and 3.58 mg/kg so, in order to homogenize the results, a new calibration/validation model for the mean of each group of six spectral measurements performed for each L_OX sample was elaborated. This way, the set of spectra was reduced from *n* = 240 to *n* = 40. Following the same procedure as previously explained, a first calibration equation was given. 

The validation model after the second preprocessing procedure (Linear Offset Subtraction), showed a Coefficient of Determination of 60.84%, a RMSECV of 0.527 and a RPD of 1.6, unlike the first results of validation, which showed a poorer R^2^ of 33.63%, a quite higher RMSCV of 0.686, and a lower RPD of 1.23. The type of validation employed was cross validation with one sample exclusion. In this case, no outlier spectra were subtracted, so a total of 40 spectra data were employed for calibration/validation model. Table 4 shows the calibration and validation results for both the entire and mean set of data.

As observed in Table 4, the most remarkable improvements appear in the calibration model when performing the mean of the group of spectral data. In that case, R^2^ increases from 75.9% to 92.74%. The root mean square error of estimation (RMSEE) decreases from 0.338 to 0.262, and the RPD goes from 2.01 to 3.71. However, the validation model shows worse results for the mean than for the whole set of spectra. This is directly related to the use of a noticeably smaller set of spectra (40 in comparison to 240), so differences between samples have stronger repercussions over the results. 

#### 3.3.2. Protein Oxidation Results

In the case of carbonyl content validation model results, R^2^_cv_ values for raw meat showed similar results than the ones obtained through the predictive model of the entire set, with values of 24.6% and 24.09%, respectively. However, when comparing these results with those obtained with the predictive model using the mean set, a significant improvement was observed, showing a R^2^_cv_ of 54.24%, which means an improvement of 29.69%. This suggests that when studying carbonyl content through MIR spectroscopy, it is preferable to perform a mean of the obtained spectra before constructing the predictive model.

In the same way as previously explained, the procedure consisted of introducing the spectral measurements into QUANT 2 software, versus their carbonyl quantitative values. A first calibration equation was given, but, in this case, the results of first validation were not consistent at all, as they presented really poor R^2^, and high RMSECV. The negative values of R^2^_cv_ were due to their low value of rank (1), as the residuals were larger than the variance in true values. Again, as happened with L_OX sets of spectra, a preprocessing treatment for the spectral data was performed. In the case of carbonyls, Derivative + Multiplicative Scatter Correction (MSC) was first performed as it offered almost the minimum RMSECV with the highest rank (5). After that, outlier points were subtracted and the validation was repeated. The validation model after the preprocessing procedure showed a R^2^ of 24.09%, a RMSECV of 0.00894 and a RPD of 1.15, unlike the first results of validation which showed a poorer R^2^ of 3.33%, a quite higher RMSCV of 0.0116, and a lower RPD of 0.984. This time, cross validation with one sample exclusion was employed. In this case, five outlier spectra were subtracted, so a total of 103 spectra were employed for the calibration/validation. The spectral range was focused on one particular area, between 1839 and 1478 cm^−1^, and the carbonyls concentration range was between 0.088 and 0.134 mM.

Following the same procedure as the one followed with the L_OX compound markers, calibration/validation models were set for the mean of the spectral measurements of P_OX compounds. Sample sets in this case were reduced from *n* = 108 to *n* = 18 to get better results compared to the analysis of the whole set of spectra of P_OX. As was expected, attending to the calibration results, the first validation outcomes were not consistent at all, as they presented the worst R^2^ values of the whole study (−24.41%), and quite high RMSECV values. As happened in all the previous analysis, a preprocessing treatment for the spectral data was done. Min-Max Normalization was performed as it offered the minimum RMSECV with an acceptable rank (8). In this case, the validation model after the preprocessing procedure of P_OX mean spectra showed a R^2^ of 54.24%, a RMSECV of 0.00769 and a RPD of 1.48. This time, cross validation with one sample exclusion validation model was chosen. In this case, no outlier spectra were subtracted, so the total spectra for calibration/validation employed were 18. The spectral range was focused between 3638 and 3277 cm^−1^. The concentration range for Carbonyls was 0.088–0.134 mM. Calibration and validation results from the entire set of spectra are presented and compared with mean sets results in Table 5.

As in L_OX results, the most remarkable improvements in the results shown at Table 5 appear in the calibration model when performing the mean of each group of spectra, and comparing the results with their entire sets. Thus, R^2^ ascends from 41.44% to 98.46% in P_OX, and as for RMSEE, it descends significantly from 0.00809 to 0.00199. Not only that, but RPD also shows a great increment in P_OX from 1.31 up to 8.06. 

In the case of validation models, P_OX results show an improvement when performing means compared to the ones obtained with the whole set of spectra. R^2^_cv_ value in the entire set increases from 24.09 to 54.24 when employing the mean spectra set. Here, the most important factor is the rank chosen. When performing preprocessing methods in the entire set of P_OX, a very low rank was chosen, something that might have affected these results. However, in the P_OX mean set, a higher rank was obtained and this could have helped to obtain more consistent results of R^2^_cv_, RPD_cv_ and RMSECV. 

#### 3.3.3. Raw Meat vs. Marker Compounds Quantitative Analysis Results

In this section, we discuss the results achieved for both type of samples, oxidation marker compounds (L_OX and P_OX), and the ones obtained in the quantitative analysis of the spectra analyzed directly from raw meat [22].

**Figure 6:**
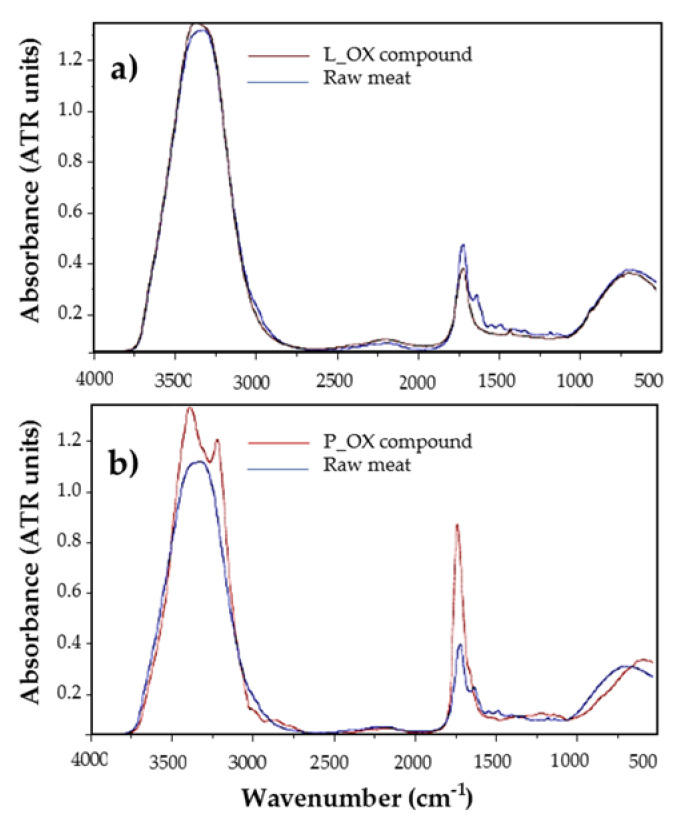
Comparison of marker compounds spectra vs. its raw meat samples: (**a**) L_OX (lipid oxidation) compound (brown) vs. raw meat animal (blue) (**b**) P_OX (protein oxidation) compound (red) vs. raw meat animal (blue).

As an approach to this comparison, some spectra are presented in Figure 6, showing L_OX and P_OX responses of some of the randomly selected samples compared to their correspondent raw meat MIR spectra. Moreover, a quantitative comparison of both groups of spectra is shown in Table 6.

As it can be observed, only slight differences can be appreciated between L_OX and raw meat samples, with both spectra being quite similar; however, raw meat spectra present several absorption peaks in the 1.650 to 1400 cm^−1^ region that L_OX samples do not show, as can be appreciated, for example, at 1336 cm^−1^. There, a weak absorption peak can be appreciated in L_OX spectra, which does not appear in raw meat samples, and it is related to the CH3 symmetric deformation and CH2 wagging group. 

Regarding the protein comparison, the differences observed in the P_OX spectral response are more significant. The main difference is the appearance of a double absorption peak in the band located between 3500 and 3000 cm^−1^ (Amide A, and Amide B group), which represents the N-H bond stretching vibrations. A higher absorption peak appears around 1650 cm^−1^, corresponding to Amide I group. This one is the most intense absorption band in proteins and is due to the stretching vibrations of the C=O (70–85%) and C-N groups (10–20%). It makes sense that these peaks present higher absorption values in the P_OX spectra than in the raw meat one, as these peaks are associated to peptide bonds. These bonds may be much more concentrated in the P_OX marker compounds than in raw meat samples, which present a much more complex tissue matrix and, hence, may interfere with the measured results.

Several differences can be found among the results observed in Table 6. Regarding L_OX extracts, very significant differences can be found between raw samples and compounds results, from calibration values of R^2^ = 9.96% in raw meat to R^2^ = 75.19% in L_OX entire set calibration (dif. = +65.23). Focusing on the validation models, the differences observed are of the same order, with a value of R^2^_cv_ = 2.43% in the raw samples, to a value of 63.18% in the entire set of L_OX samples. In the rest of the studied parameters, the results are less consistent in raw meat samples than the ones obtained through L_OX compounds through MIR analysis. It should also be noted that the highest RPD value of the whole group of predictive models was obtained through the L_OX entire set, achieving a value of 1.65.

On the other hand, the results found of the entire set and the mean set of carbonyls are strikingly different in the case of P_OX. Concretely, in the validation models, R^2^ in raw meat samples showed a very similar result to the one obtained through the predictive model of the entire set of P_OX, with values of 24.6% and 24.09%, respectively. However, when comparing it to the result obtained through the predictive model of the mean set, a significant improvement can be observed in the latter one, with a R^2^_cv_ = 54.24% (diff. = +29.69). This may suggest that when studying P_OX content through MIR spectroscopy, it is preferable to perform a mean of the spectra obtained before constructing the predictive model.

## 4. Conclusions

The spectra showed some remarkable differences between protein and lipid oxidation marker compounds. Prediction models were promising for the usefulness of MIR technology to estimate the values of lipid and protein oxidation marker compounds from previously extracted samples or when applied directly on raw meat samples. However, marker compound results showed more consistent predictive models than the ones achieved using quantitative analysis of the spectra obtained from the raw meat. From a practical point of view, given the results, the use of regression treatments delimiting the range of the spectrum coinciding with the wavelengths in which a large number of protein and fatty acid bonds are recognized could be useful in future research. Besides, it has been proved that performing a previous extraction of the marker compounds notably helps to achieve more consistent results for lipid and protein oxidation assessment in horse meat products. Considering the potential of this technique, MIR spectroscopy arises as an interesting alternative to traditional meat composition analytical methods, which are time consuming. Regardless, more research is needed on the MIR application directly on raw meat in order to improve the estimation of the obtained predictive models. 

## Figures and Tables

**Table 1:** Lipid and protein oxidation quantification of Young-Linseed (Y-L) and Adult Conventional (A-C) foal meat.

	TBA (MDA/kg)	Total Carbonyl (nmole/mg)
Y-L 0d	0.4525 ^a^	4.07 ^a^
Y-L 4d	0.4387 ^a^	4.20 ^ab^
Y-L 8d	0.505 ^a^	4.35 ^ab^
Y-L 12d	0.5287 ^a^	4.28 ^ab^
A-C 4d	2.1042 ^b^	4.68 ^b^
SEM	0.054	0.059
*p*-VALUE	0.000	0.009

TBA: Thiobarbituric Acid; Means within a column lacking a common superscript letter differ (*p* < 0.05).

**Table 2:** Compilation of principal wavenumbers associated to functional groups.

Wavenumber (cm^−1^)	Assignment	Reference
3700–3200	Stretching vibration of bonded and non-bonded -O-H groups (water vibration)	[24]
3500–3300	Amines	[25]
~3400	Amide A (N-H stretching vibrations)	[25]
~3100	Amide B (N-H stretching vibrations)	[26]
3000–2850	Symmetrical and asymmetrical stretching vibrations of C-H groups (Alkanes); fatty acids backbone	[27]
1659–1653	=C-H stretching vibrations/Amide I (C=O stretching vibration)/O-H bending vibrations in water	[25,28]
1680–1620	Stretching vibration of C=H groups (Alkenes)	[29]
~1640	Hydrogen covalent bonds bending of water	[30]
1580–1540	C-O vibrations/Amide II (N-H bending vibration mixed with C-N stretching vibration)/Aromatic -C=C stretching vibrations	[31]
1400–1200	Amide III (N-H, C-C and C-N vibrations)	[26]
970–920	Trans = C-H out-of-plane bending	[32]
610–711	Amide V (C-N and N-H vibrations)	[26]
900–400	Stretching of O-H (water vibration)	[24]

**Table 3:** Results of the predictive models (calibration and validation), of the TBARS, carbonyls studied in raw meat and L_OX (lipid oxidation) and P_OX (protein oxidation) marker compounds.

Parameter		TBARS Raw Meat Samples	Carbonyl Raw Meat Samples	L_OX Entire Set	L_OX Mean Set	P_OX Entire Set	P_OX Mean Set
Calibration	R^2^	9.96	94.99	75.19	92.74	41.44	98.46
	RMSEE	0.143	0.136	0.338	0.262	0.00809	0.00199
	RPD	1.05	4.47	2.01	3.71	1.31	8.06
	Rank	1	8	10	9	5	8
Validation	R^2^_cv_	2.43	24.6	63.18	60.84	24.09	54.24
	RMSECV	0.146	0.492	0.402	0.527	0.00894	0.00769
	RPD_cv_	1.01	1.15	1.65	1.6	1.15	1.48
	Rank_cv_	1	8	10	9	5	8
Preprocessing Treatment	First Deriv. + Vectorial Normalization (SNV) (2559–2199 cm^−1^)	Second Deriv. (3998–3637; 3278–2918; 2559–478cm^−1^)	SNV Normalization 3278–2918 cm^−1^	Linear Offset Subtraction Between 3998–3637 cm^−1^	MSC 1839–1478 cm^−1^	Min-Max Normalization 3638–3277 cm^−1^	Straight Line Subtraction 3278–2918; 2198–1118; 760–399 cm^−1^
Outliers (spectra)	6	3	13	0	5	0	0

RPD: Ratio of (standard error of) Prediction to (standard) Deviation. R^2^: proportion of the variance for a dependent variable that is explained by the independent variable in the regression model. RMSEE: root mean square error of estimation. RMSECV: root mean square error of cross validation.

**Table 4:** Calibration and cross validation results for entire and mean sets in the L_OX (lipid oxidation) spectra.

Parameter		L_OX Entire Set (*n* = 240)	L_OX Mean Set (*n* = 40)
Calibration	R^2^	75.19	92.74
	RMSEE	0.338	0.262
	RPD	2.01	3.71
	Rank	10	9
Validation	R^2^_cv_	63.18	60.84
	RMSECV	0.402	0.527
	RPD_cv_	1.65	1.6
	Rank_cv_	10	9

**Table 5:** Calibration and cross validation results for entire and mean sets in P_OX (protein oxidation) spectra.

		P_OX Entire Set (*n* = 108)	P_OX Mean Set (*n* = 18)
Calibration	R^2^	41.44	98.46
	RMSEE	0.00809	0.00199
	RPD	1.31	8.06
	Rank	5	8
Validation	R^2^_cv_	24.09	54.24
	RMSECV	0.00894	0.00769
	RPD_cv_	1.15	1.48
	Rank_cv_	5	8

**Table 6:** Results of the predictive models (calibration and validation), of the TBA, and carbonyls content studied in raw meat and L_OX (lipid oxidation) and P_OX (protein oxidation) markers compounds.

	Calibration	Validation							Preprocessing Treatment	Outliers (Spectra)
Parameter	R^2^	RMSEE	RPD	Rank	R^2^_cv_	RMSECV	RPD_cv_	Rank_cv_		
TBA Raw Meat Samples	9.96	0.143	1.05	1	2.43	0.146	1.01	1	First Deriv. + Vectorial Normalization (SNV) (2559–2199 cm^−1^)	6
Carbonyl Raw Meat Samples	94.99	0.136	4.47	8	24.6	0.492	1.15	8	Second Deriv. (3998–3637; 3278–2918; 2559–478 cm^−1^)	3
L_OX Entire Set	75.19	0.338	2.01	10	63.18	0.402	1.65	10	SNV Normalization (3278–2918 cm^−1^)	13
L_OX Mean Set	92.74	0.262	3.71	9	60.84	0.527	1.6	9	Linear Offset Subtraction (Between 3998–3637 cm^−1^)	0
P_OX Entire set	41.44	0.00809	1.31	5	24.09	0.00894	1.15	5	MSC (1839–1478 cm^−1^)	5
P_OX Mean Set	98.46	0.00199	8.06	8	54.24	0.00769	1.48	8	Min-Max Normalization (3638–3277 cm^−1^)	0

## Appendix A

**Table A1:** Concentrates Composition.

	Standard Concentrate			Linseed Concentrate		
	Ration (%)	Digestible Energy (Mcal/kg DM)	% Brute Protein/kg DM	Ration (%)	Digestible Energy (Mcal/kg DM)	% Brute Protein/kg DM
Ingredients						
Oat flour	30.03	2.5	7.4	45.95	2.5	7.4
Barley flour	30.10	2.9	8.3	13.07	2.9	8.3
Cornmeal	15.47	3.0	6.5	14.54	3.0	6.5
Wheat Bran	7.95	2.3	13.0	5.92	2.3	13.0
Soybean meal	9.99	3.0	38.2	9.06	3.0	38.2
Glycerol	3.96	3.2		3.96	3.2	
Linseed				5.01	3.9	19.1
TOTAL	97.50 ^1^	2.69	10.57	97.51 ^1^	2.70	10.61

^1^ 2.5 % added to the total ration addition (%) (vitamins + salt + calcium carbonate + calcium phosphate).
